# Supplementary material for: Combining genetic resources and elite material populations to improve the accuracy of genomic prediction in apple
Source: G3 (Bethesda). 2021 Dec 10;12(3):jkab420. doi: 10.1093/g3journal/jkab420 (PMC9210277; doi:10.1093/g3journal/jkab420)
Supplement: jkab420_Supplemental_Figure_Legends [file jkab420_supplemental_figure_legends.docx]

**Figure S1**. Distribution of the Allelic R² (AR²) values obtained after imputation for the elite material and the hybrids

**Figure S2.** Linkage disequilibrium decay for SNPs within a 500kb distance in the elite material and genetic resources of the FBo-Hi and REFPOP datasets

**Figure S3.** Frequence of the minor allele in the genetic resources and of the same allele in the elite material along the 17 chromosomes of the apple genome in the FBo-Hi dataset. Allele frequencies are computed using sliding windows of 2Mb with a shift of 400kb.

**Figure S4.** Frequence of the minor allele in the genetic resources and of the same allele in the elite material along the 17 chromosomes of the apple genome in the REFPOP dataset. Allele frequencies are computed using sliding windows of 2Mb with a shift of 400kb.

**Figure S5.** Phenotypic distribution of the traits measured in the FBo-Hi dataset only. **A**. Acidity **B.** Juiciness **C.** Crispness

**Figure S6.** Phenotypic distribution of the traits measured in the REFPOP dataset and the dataset of hybrids. **A**. Fruit number **B.** Fruit weight

**Figure S7.** Phenotypic distribution of the traits measured in the dataset of hybrids and FBo-Hi and REFPOP datasets. **A**. Harvest date **B.** Fruit over-color

**Figure S8.** Broad-sense heritability for each site and year in the REFPOP dataset. **BEL** Belgium **CHE** Switzerland **ESP** Spain **FRA** France **ITA** Italy

**Figure S9.** Predictive abilities for acidity in the FBo-Hi dataset with medium and high marker density. **WP**: within-population prediction **AP**: across-population prediction **Comb**: combination prediction **MG-Comb**: combination prediction with the MG-GBLUP method

**Figure S10.** Predictive abilities for crispness in the FBo-Hi dataset with medium and high marker density. **WP**: within-population prediction **AP**: across-population prediction **Comb**: combination prediction **MG-Comb**: combination prediction with the MG-GBLUP method

**Figure S11.** Predictive abilities for juiciness in the FBo-Hi dataset with medium and high marker density. **WP**: within-population prediction **AP**: across-population prediction **Comb**: combination prediction **MG-Comb**: combination prediction with the MG-GBLUP method

**Figure S12.** Predictive abilities for fruit number in the REFPOP dataset with medium and high marker density. **WP**: within-population prediction **AP**: across-population prediction **Comb**: combination prediction **MG-Comb**: combination prediction with the MG-GBLUP method

**Figure S13.** Predictive abilities for fruit weight in the REFPOP dataset with medium and high marker density. **WP**: within-population prediction **AP**: across-population prediction **Comb**: combination prediction **MG-Comb**: combination prediction with the MG-GBLUP method

**Figure S14.** Predictive abilities for fruit number in the dataset of hybrids with medium and high marker density when the training set is composed of varying proportions of elite material and genetic resources of the REFPOP dataset. **max(predAbi)**: maximum predictive ability obtained for a given marker density with one of the three tested methods regardless of the training set size **Random**: predictive ability obtained when randomly choosing the genotypes included in the training set

**Figure S15.** Predictive abilities for fruit weight in the dataset of hybrids with medium and high marker density when the training set is composed of varying proportions of elite material and genetic resources of the REFPOP dataset. **max(predAbi)**: maximum predictive ability obtained for a given marker density with one of the three tested methods regardless of the training set size **Random**: predictive ability obtained when randomly choosing the genotypes included in the training set

**Figure S16**. Evolution of the predictive ability for harvest date in the FBo-Hi dataset when the candidates are predicted using an increasing TS size from the same population (WP_inc_), all the genotypes from the complementary population (AP) or a combination of both populations (Comb_inc_).

**Figure S17**. Evolution of the predictive ability for fruit over-color in the FBo-Hi dataset when the candidates are predicted using an increasing TS size from the same population (WP_inc_), all the genotypes from the complementary population (AP) or a combination of both populations (Comb_inc_).

**Figure S18**. Evolution of the predictive ability for acidity in the FBo-Hi dataset when the candidates are predicted using an increasing TS size from the same population (WP_inc_), all the genotypes from the complementary population (AP) or a combination of both populations (Comb_inc_).

**Figure S19**. Evolution of the predictive ability for crispness in the FBo-Hi dataset when the candidates are predicted using an increasing TS size from the same population (WP_inc_), all the genotypes from the complementary population (AP) or a combination of both populations (Comb_inc_).

**Figure S20**. Evolution of the predictive ability for juiciness in the FBo-Hi dataset when the candidates are predicted using an increasing TS size from the same population (WP_inc_), all the genotypes from the complementary population (AP) or a combination of both populations (Comb_inc_).

**Figure S21**. Comparison between the GEBV obtained using the GBLUP or MG-GBLUP models for crispness. The correlation between the GEBV obtained using the two methods for a given population and genomic density is presented in each box.

**Figure S22**. Comparison between the GEBV obtained using the GBLUP or MG-GBLUP models for fruit weight. The correlation between the GEBV obtained using the two methods for a given population and genomic density is presented in each box.

**Figure S23**. Principal Component Analysis (PCA) performed using pruned marker data of the REFPOP and hybrids panel.
