# Supplementary material for: Combining genetic resources and elite material populations to improve the accuracy of genomic prediction in apple
Source: G3 (Bethesda). 2021 Dec 10;12(3):jkab420. doi: 10.1093/g3journal/jkab420 (PMC9210277; doi:10.1093/g3journal/jkab420)
Supplement: jkab420_Supplemental_Tables [file jkab420_supplemental_tables.docx]

**Table S1**. Mating design and family sizes of the hybrids panel

|  |  | Genetic resources | | | | |
| --- | --- | --- | --- | --- | --- | --- |
|  |  | X08233 | X08483 | X02353 | X02640 | X08488 |
| Elite | X02437 | 65 |  |  |  |  |
|  | X03263 |  |  |  | 39 |  |
|  | X03318 |  |  |  |  | 49 |
|  | X06407 |  | 38 |  |  |  |
|  | X06683 |  |  | 50 |  |  |
|  | X06963 | 31 |  |  |  |  |
|  | X07860 | 51 |  |  |  |  |
|  | FRAi0085 | 44 | 50 |  |  |  |
|  | X08486 | 56 |  |  |  |  |

**Table S2**. Year range and heritability of the traits measured in the FBo-Hi dataset

|  | **CRA-W** | | **INRA** | | **NFC** | | **RBIPH** | | **SLU** | | **UNIBO** | |
| --- | --- | --- | --- | --- | --- | --- | --- | --- | --- | --- | --- | --- |
|  | No. years (range) | H² | No. years (range) | H² | No. years (range) | H² | No. years (range) | H² | No. years (range) | H² | No. years (range) | H² |
| Acidity | 13 (1986-2014) | 0.90 | 8 (2002-2014) | 0.80 | 1 | - | 2 (2006-2010) | 0.90 | 3 (2012-2014) | 0.90 | 5 (1988-2014) | 0.7 |
|  |  |  |  |  |  |  |  |  |  |  |  |  |
| Crispness | 8 (1987-2013) | 0,80 | 4 (2010-2014) | 0.70 | 2 (2012-2013) | 0.59 | 3 (2012-2014) | 0.72 | 2 (2012-2014) | 0.97 | 3 (2012-2014) | 0.5 |
|  |  |  |  |  |  |  |  |  |  |  |  |  |
| Juiciness | 12 (1986-2013) | 0,76 | 7 (2004-2014) | 0.70 | 2 (2012-2013) | 0.58 | 5 (2006-2010) | 0.74 | 3 (2012-2014) | 0.89 | 3 (2012-2014) | 0.6 |
|  |  |  |  |  |  |  |  |  |  |  |  |  |
| Fruit over-color | 4 (1989-2013) | 0,90 | 7 (2002-2010) | 0.81 | 1 | - | 5 (2006-2010) | 0.93 | 3 (2012-2014) | 0.94 | 9 (1987-2014) | 0.94 |
|  |  |  |  |  |  |  |  |  |  |  |  |  |
| Harvest date | 1 (2013) | - | 3 (2012-2014) | 0.96 | 3 (2012-2014) | 0.88 | - | - | 3 (2012-2014) | 0.98 | 3 (2012-2014) | 0.97 |
|  |  |  |  |  |  |  |  |  |  |  |  |  |

**Table S3**. Pairwise F_ST_ values computed from marker data for the elite material, genetic resources and hybrids in the two datasets

| FBo-Hi dataset | | |
| --- | --- | --- |
| Population | Elite material | Genetic resources |
| Genetic resources | 0.023 | - |
| Hybrids | 0.029 | 0.017 |
| REFPOP dataset | | |
| Population | Elite material | Genetic resources |
| Genetic resources | 0.021 | - |
| Hybrids | 0.029 | 0.017 |
