# Supplementary material for: Combining genetic resources and elite material populations to improve the accuracy of genomic prediction in apple
Source: G3 (Bethesda). 2021 Dec 10;12(3):jkab420. doi: 10.1093/g3journal/jkab420 (PMC9210277; doi:10.1093/g3journal/jkab420)
Supplement: jkab420_Supplementary_Material_1 [file jkab420_supplementary_material_1.docx]

# Supplementary Material 1

## Effect of the training set size on predictive ability

For the elite material and genetic resources of the FBo-Hi dataset, we evaluated the impact of the number of genotypes in the TS on predictive ability for the within-population prediction scenario. To do so, we used the 100 sets of candidates used in the WP scenario (coming from 20 replications of a fivefold cross-validation scheme) and predicted each set with a training set of randomly chosen *x* individuals from the remaining genotypes of the same population, where *x* starts at 50 genotypes and is increased by steps of 50 genotypes until all the available genotypes are part of the training set. For each training set constituted this way, the predictive ability was evaluated as the Pearson correlation coefficient between the GEBV and the phenotypic values of the individuals in the validation set. In a second step, we added all the genotypes from the complementary population to the x genotypes of the training set. We call the first scenario WP_inc_ and the second one Comb_inc_. Note that the WP and Comb scenarios correspond to the WP_inc_ and Comb_inc_ scenario when the value of x corresponds to the maximum number of genotypes in the training set.

For the Comb_inc_ scenario, the MG-GBLUP model was not evaluated because of the high computational time it would require to apply the model for each value of *x*.
