# Supplementary material for: Combining genetic resources and elite material populations to improve the accuracy of genomic prediction in apple
Source: G3 (Bethesda). 2021 Dec 10;12(3):jkab420. doi: 10.1093/g3journal/jkab420 (PMC9210277; doi:10.1093/g3journal/jkab420)
Supplement: jkab420_Supplementary_Figures [file jkab420_supplementary_figures.zip › jkab420_Supplementary_Figures/Figure_S17.pdf]

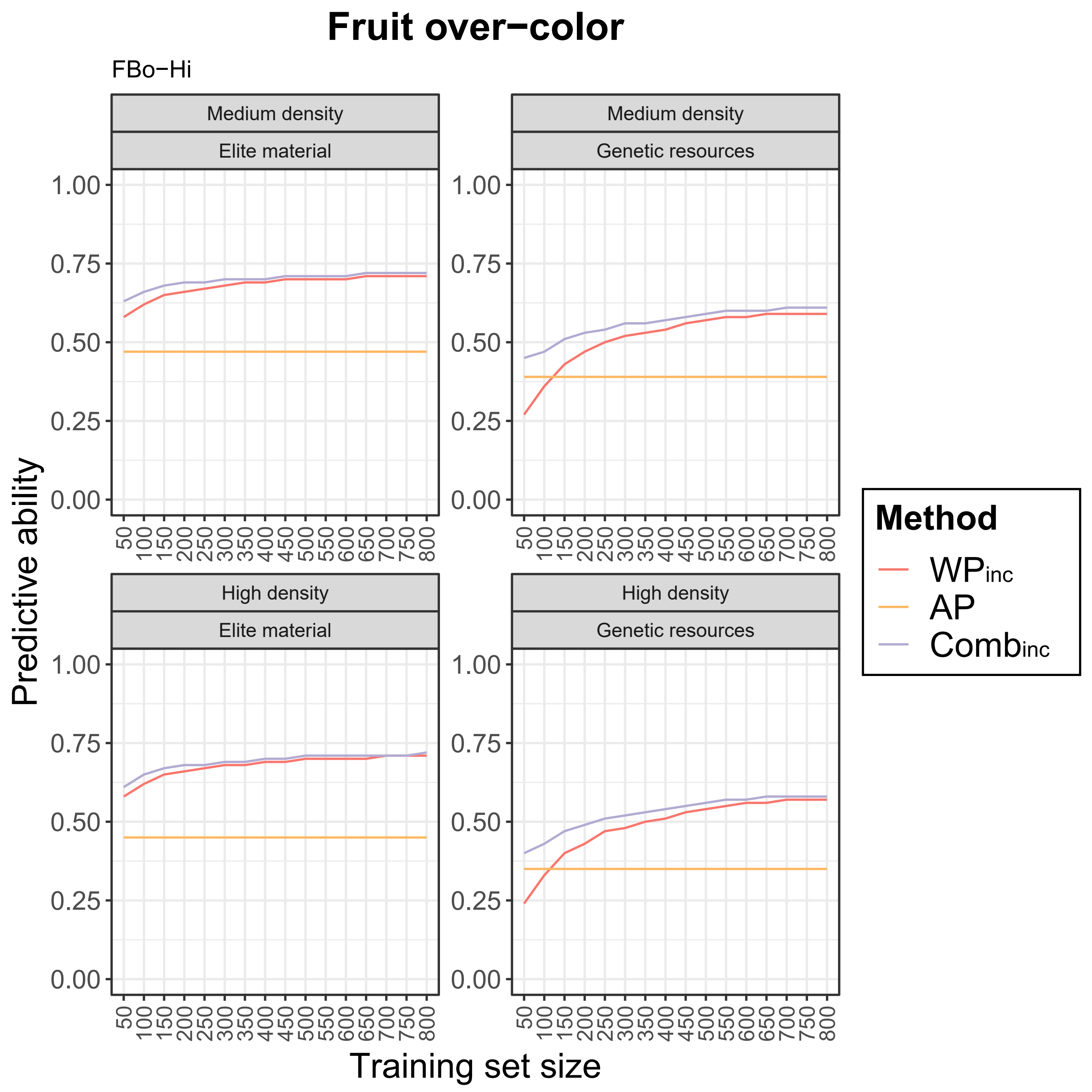

Fruit over−color
FBo−Hi
Medium density
Elite material
Genetic resources
High density
1.00
0.75
0.50
0.25
0.00
50
100
150
200
250
300
350
400
450
500
550
600
650
700
750
800
Predictive ability
Training set size
Method
WPinc
AP
Combinc
