# Supplementary material for: Combining genetic resources and elite material populations to improve the accuracy of genomic prediction in apple
Source: G3 (Bethesda). 2021 Dec 10;12(3):jkab420. doi: 10.1093/g3journal/jkab420 (PMC9210277; doi:10.1093/g3journal/jkab420)
Supplement: jkab420_Supplementary_Figures [file jkab420_supplementary_figures.zip › jkab420_Supplementary_Figures/Figure_S21.pdf]

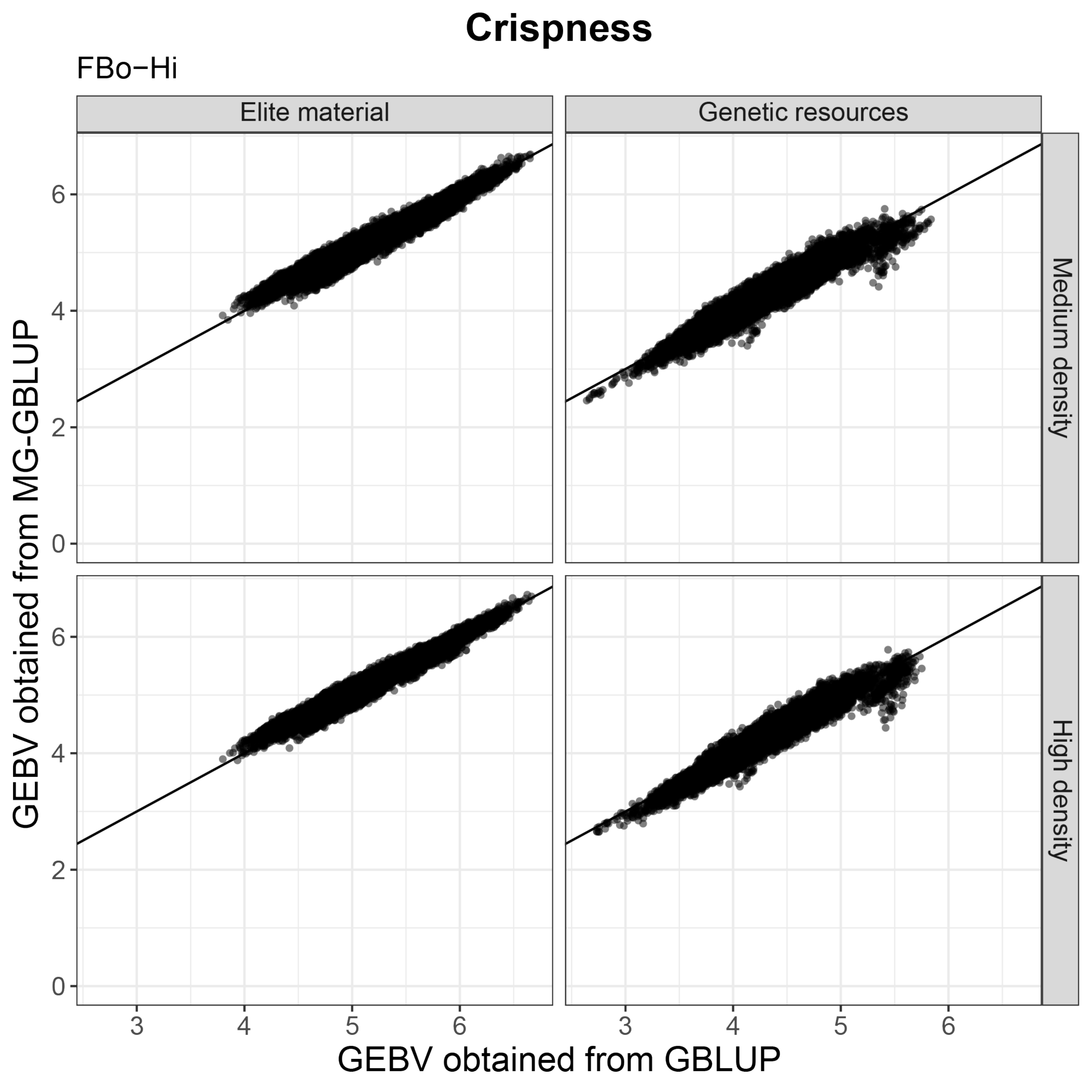
Crispness
FBo−Hi
Elite material
Genetic resources
Medium density
High density
GEBV obtained from MG-GBLUP
GEBV obtained from GBLUP
0
2
4
6
3
4
5
6
