# Supplementary figures and images for: Combining genetic resources and elite material populations to improve the accuracy of genomic prediction in apple
Source: G3 (Bethesda). 2021 Dec 10;12(3):jkab420. doi: 10.1093/g3journal/jkab420 (PMC9210277; doi:10.1093/g3journal/jkab420)

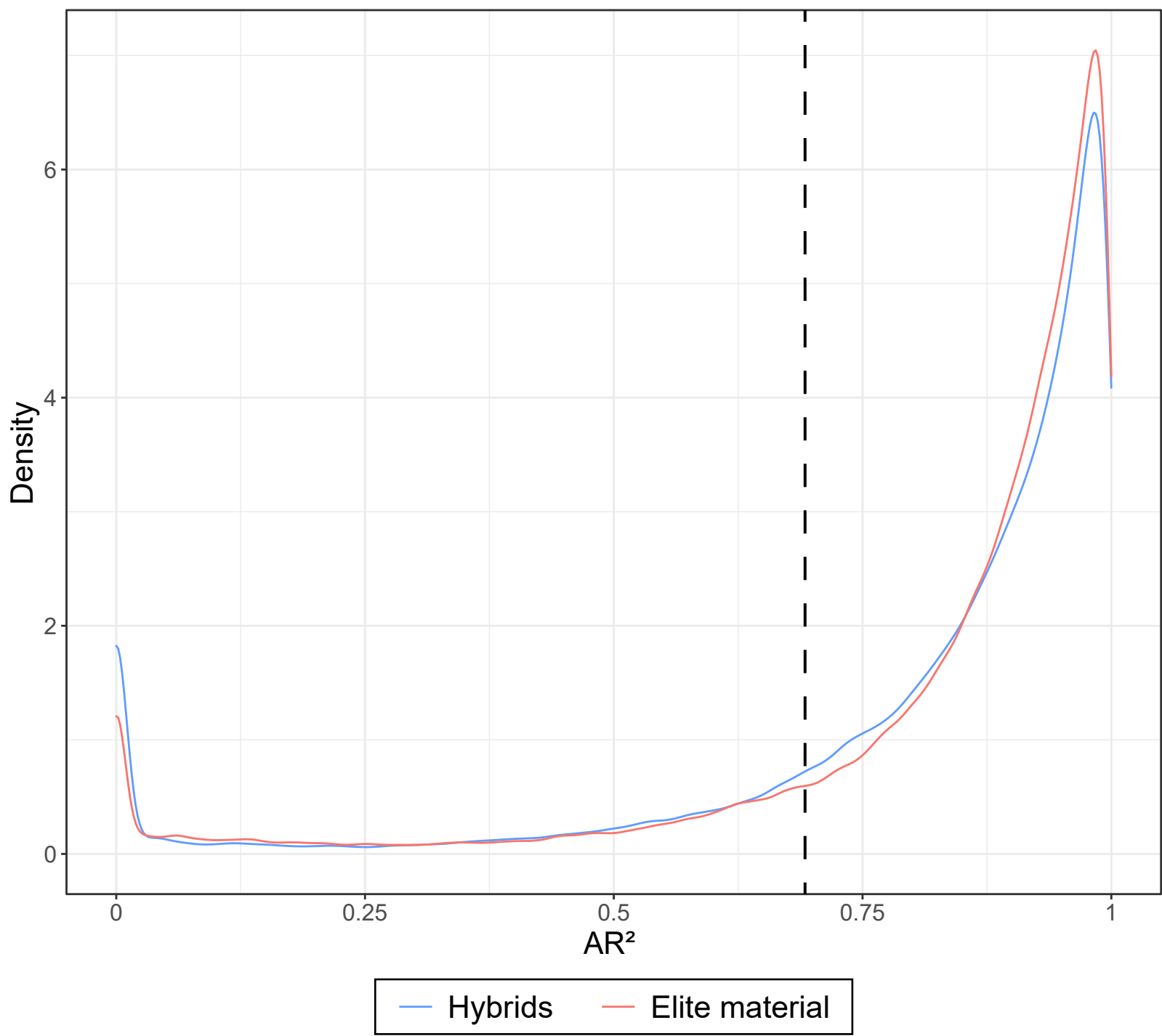

Supplement: jkab420_Supplementary_Figures [file jkab420_supplementary_figures.zip › jkab420_Supplementary_Figures/Figure_S1.pdf]

# Crispness

Fbo-Hi

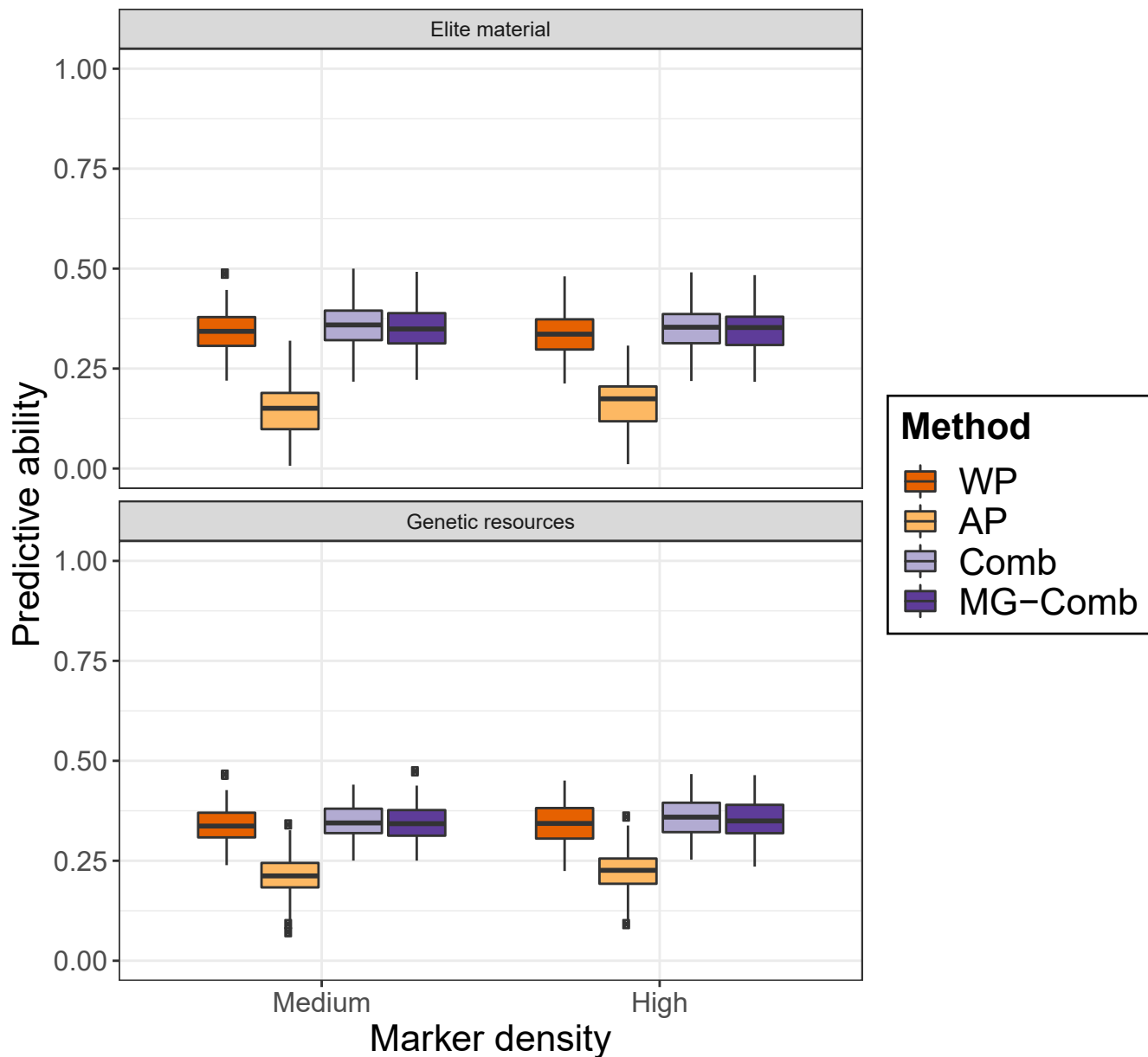

Supplement: jkab420_Supplementary_Figures [file jkab420_supplementary_figures.zip › jkab420_Supplementary_Figures/Figure_S10.pdf]

# Juiciness

Fbo-Hi

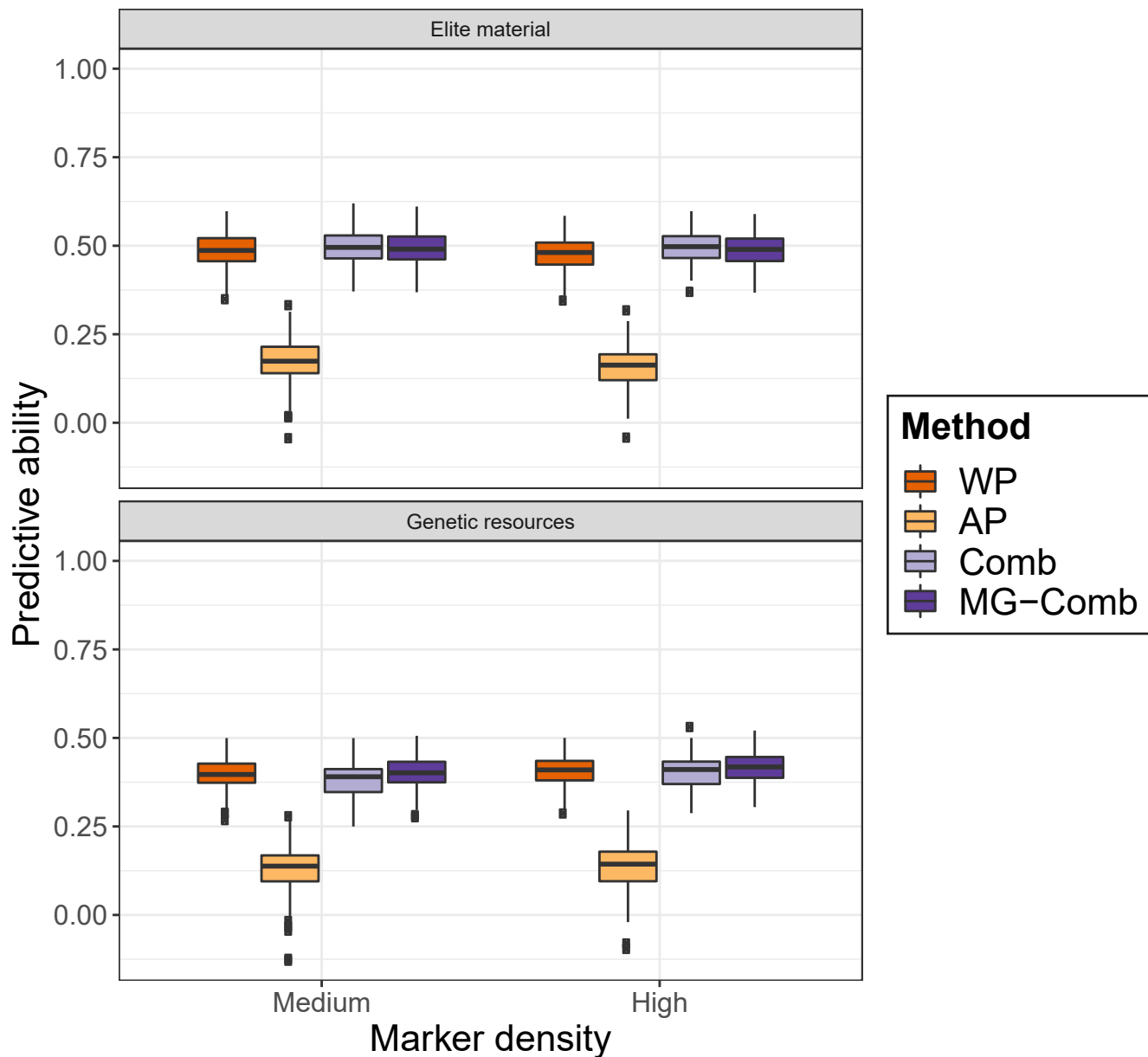

Supplement: jkab420_Supplementary_Figures [file jkab420_supplementary_figures.zip › jkab420_Supplementary_Figures/Figure_S11.pdf]

# Fruit number

REFPOP

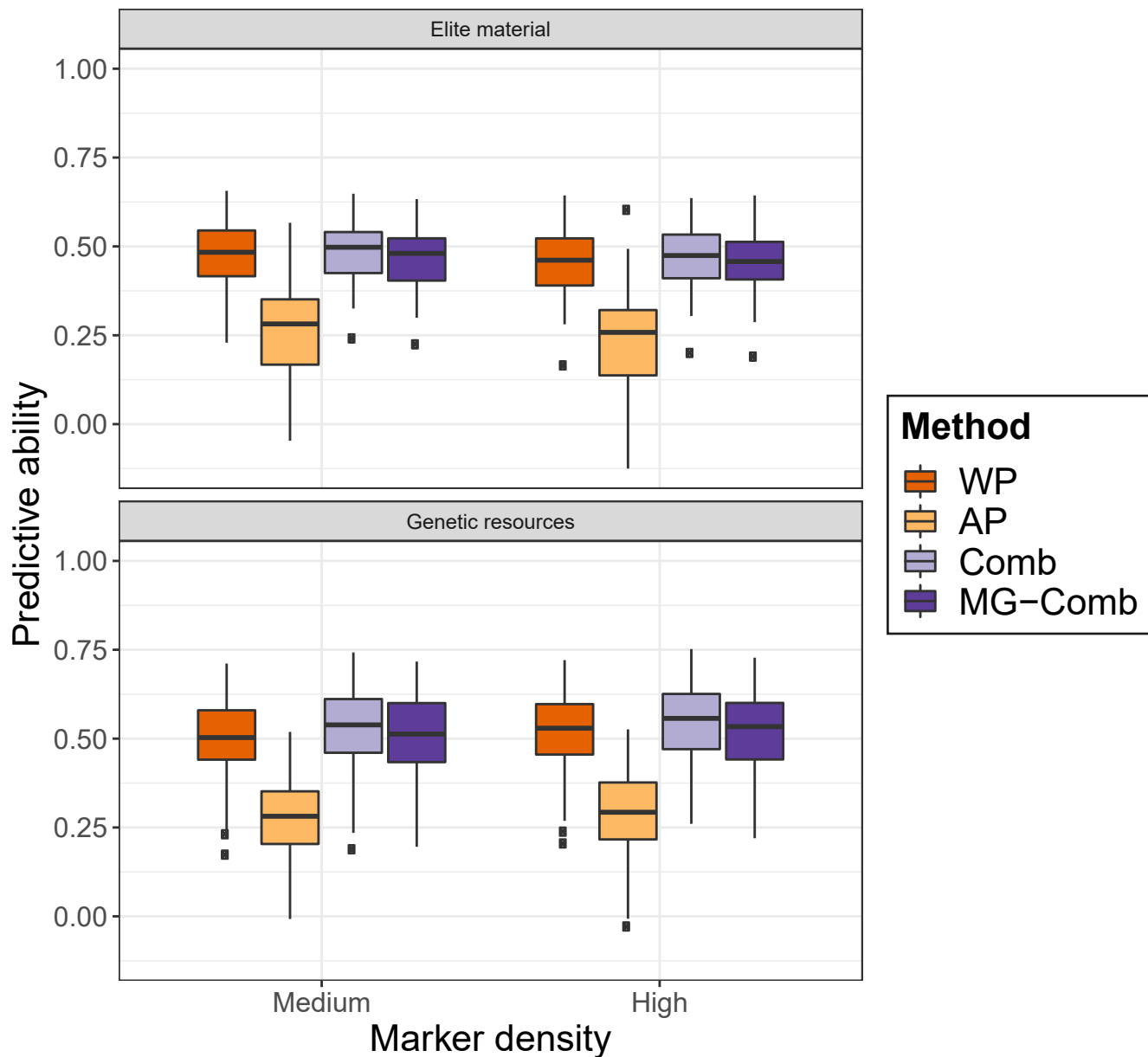

Supplement: jkab420_Supplementary_Figures [file jkab420_supplementary_figures.zip › jkab420_Supplementary_Figures/Figure_S12.pdf]

# Fruit weight

REFPOP

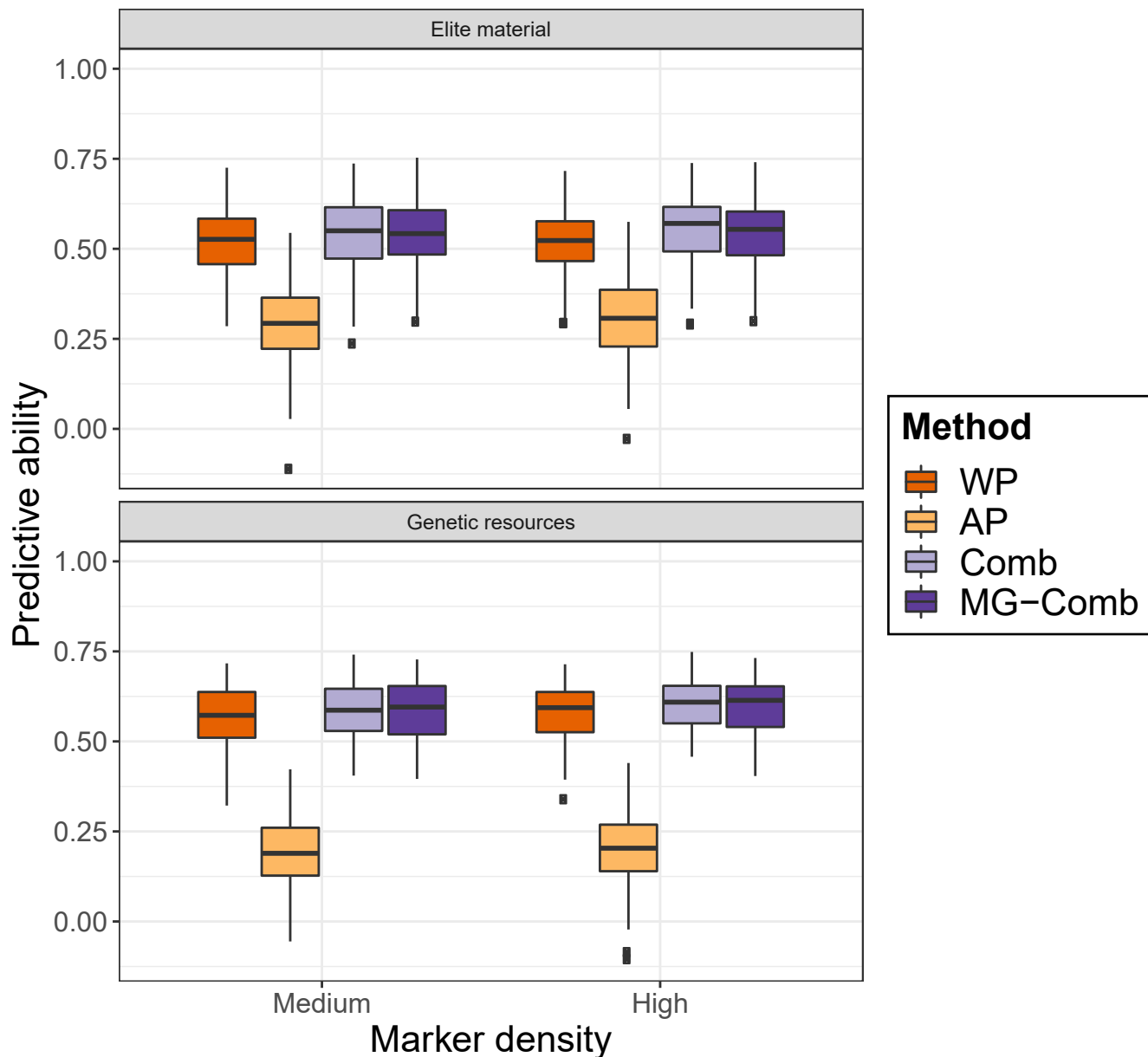

Supplement: jkab420_Supplementary_Figures [file jkab420_supplementary_figures.zip › jkab420_Supplementary_Figures/Figure_S13.pdf]

# Fruit number

REFPOP

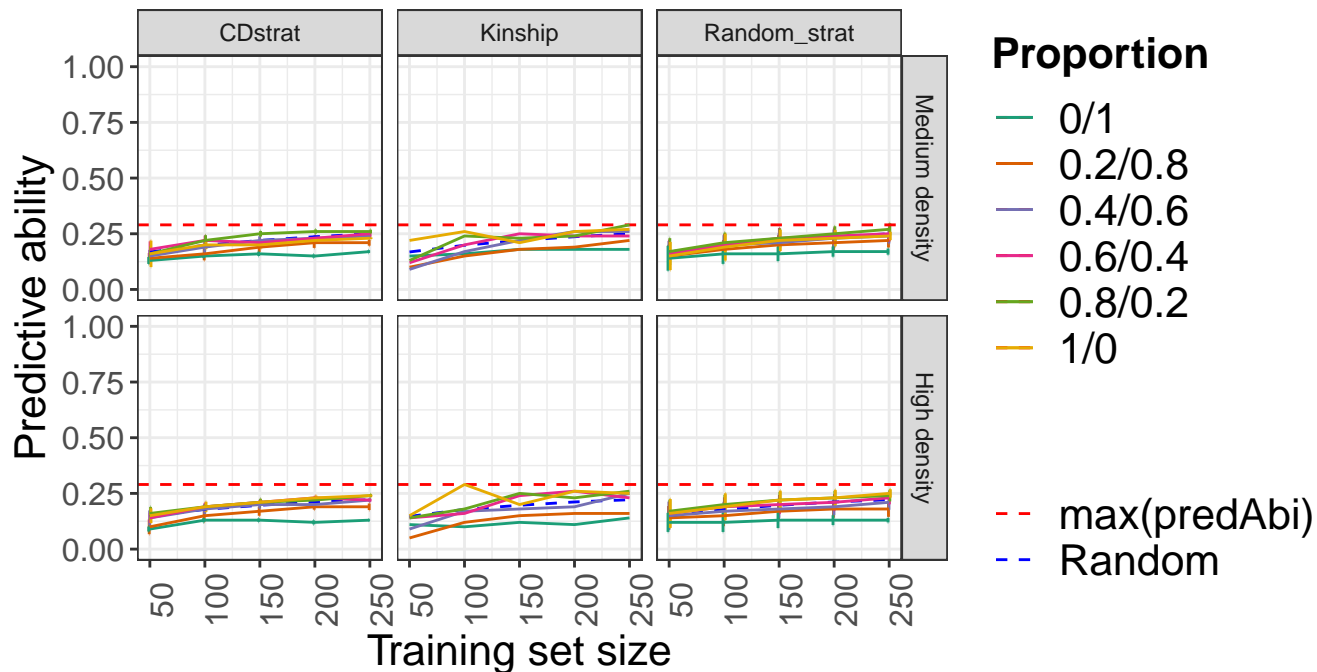

Supplement: jkab420_Supplementary_Figures [file jkab420_supplementary_figures.zip › jkab420_Supplementary_Figures/Figure_S14.pdf]

# Fruit weight

REFPOP

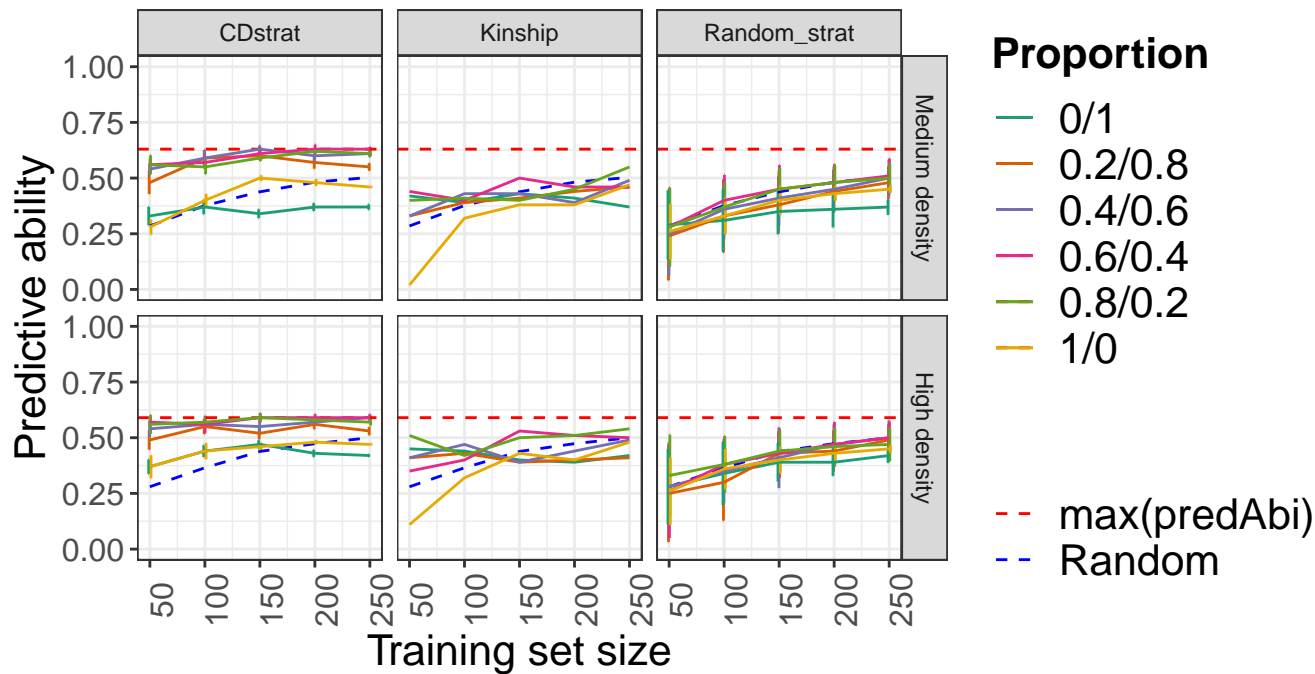

Supplement: jkab420_Supplementary_Figures [file jkab420_supplementary_figures.zip › jkab420_Supplementary_Figures/Figure_S15.pdf]

# Harvest date

FBo-Hi

Predictive ability

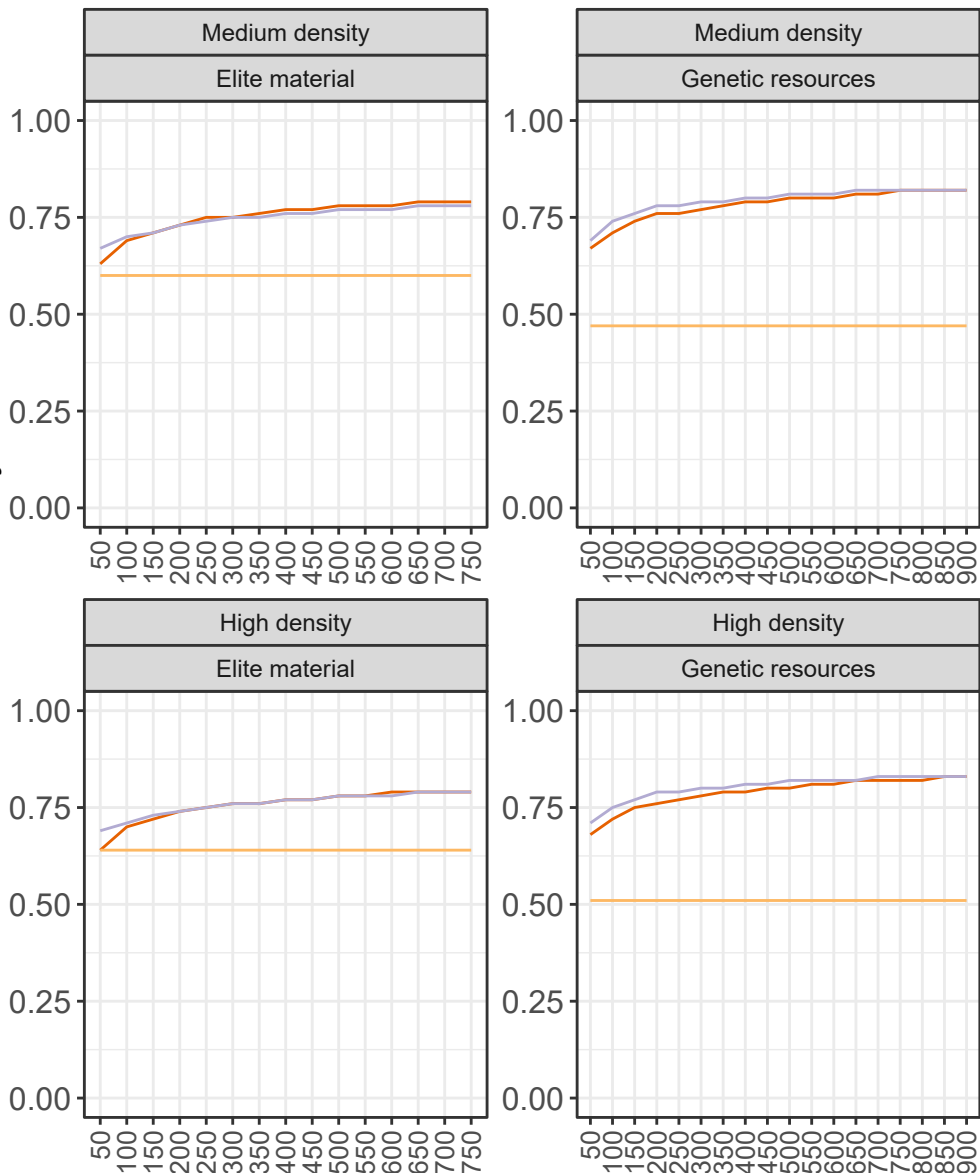

Method

- $WP_{inc}$
- AP
- $Comb_{inc}$

Training set size

Supplement: jkab420_Supplementary_Figures [file jkab420_supplementary_figures.zip › jkab420_Supplementary_Figures/Figure_S16.pdf]

# Acidity

FBo-Hi

Predictive ability

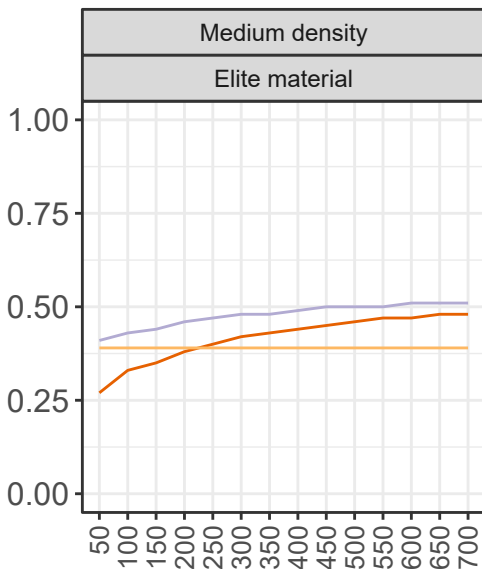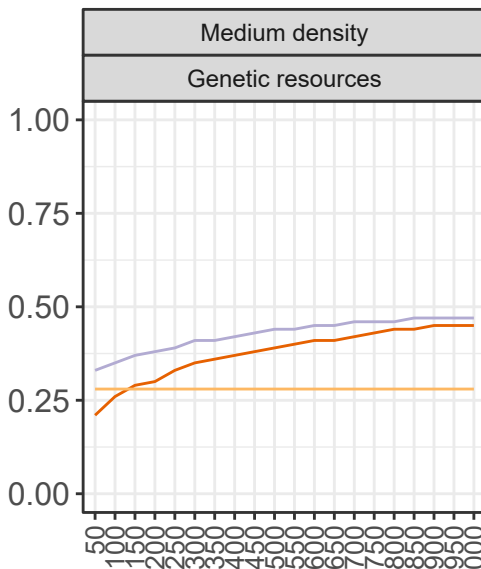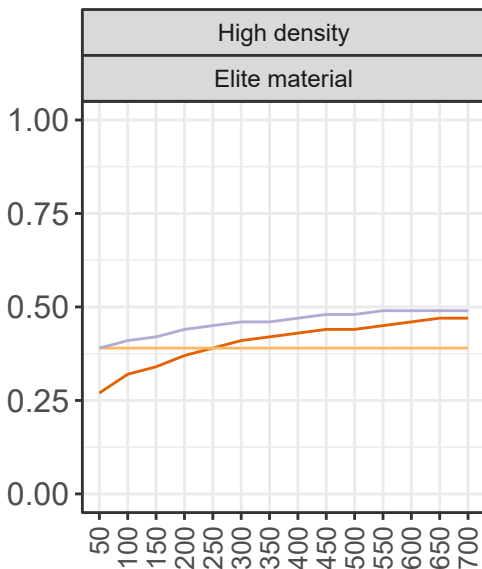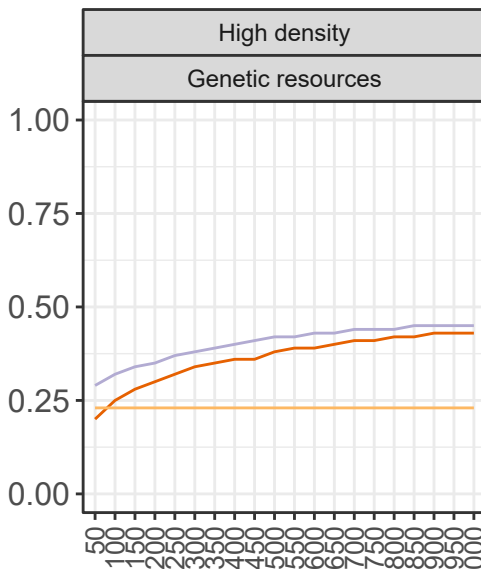

**Method**

- WP<sub>inc</sub>
- AP
- Comb<sub>inc</sub>

Training set size

Supplement: jkab420_Supplementary_Figures [file jkab420_supplementary_figures.zip › jkab420_Supplementary_Figures/Figure_S18.pdf]

# Crispness

FBo-Hi

Predictive ability

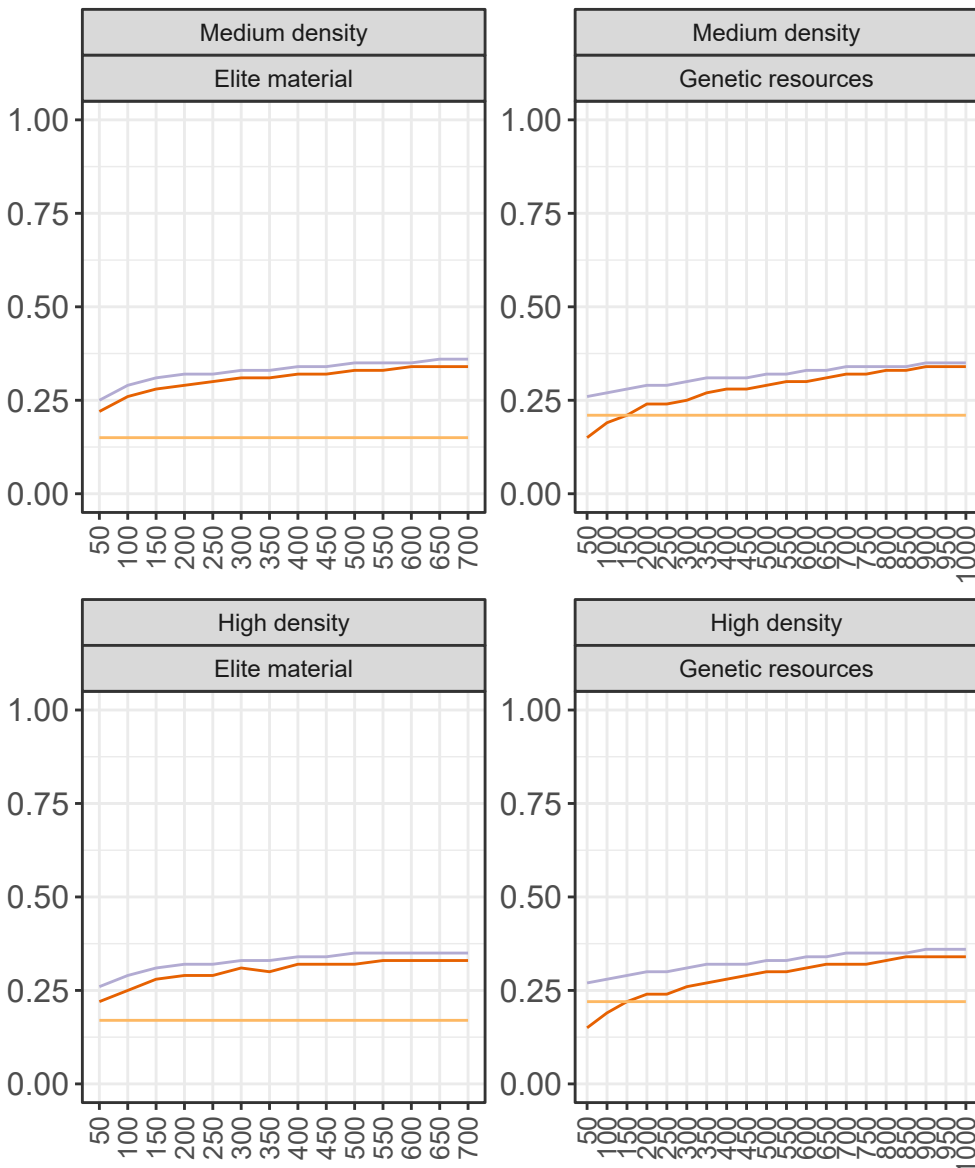

**Method**

- WP<sub>inc</sub>
- AP
- Comb<sub>inc</sub>

Training set size

Supplement: jkab420_Supplementary_Figures [file jkab420_supplementary_figures.zip › jkab420_Supplementary_Figures/Figure_S19.pdf]

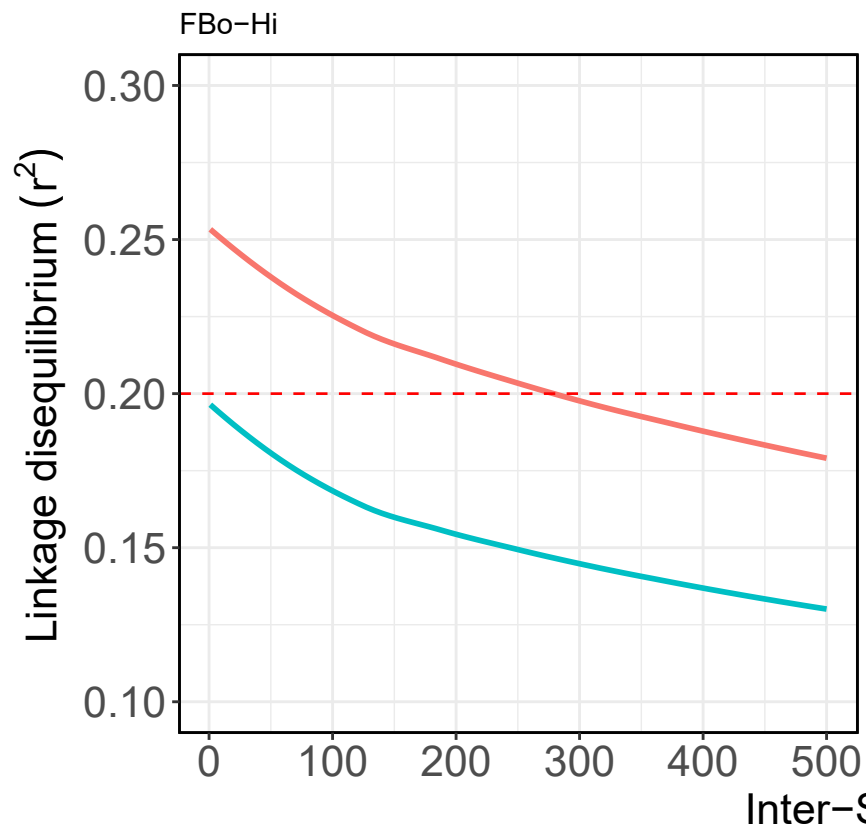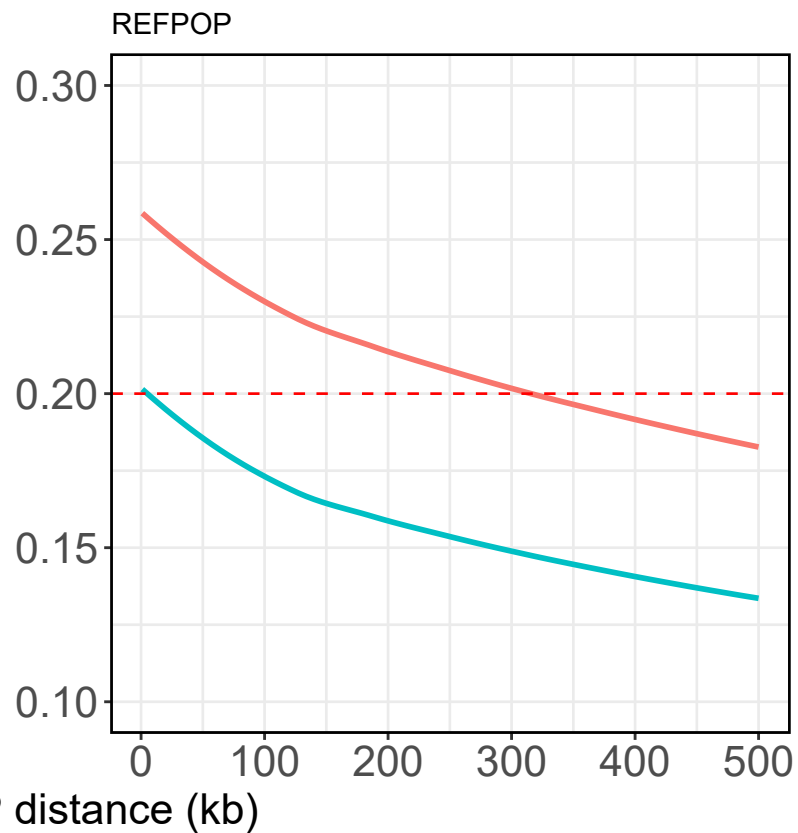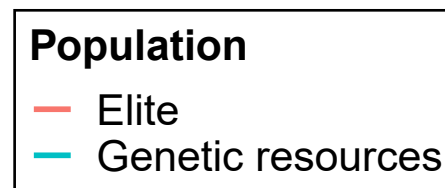

Supplement: jkab420_Supplementary_Figures [file jkab420_supplementary_figures.zip › jkab420_Supplementary_Figures/Figure_S2.pdf]

# Juiciness

FBo-Hi

Predictive ability

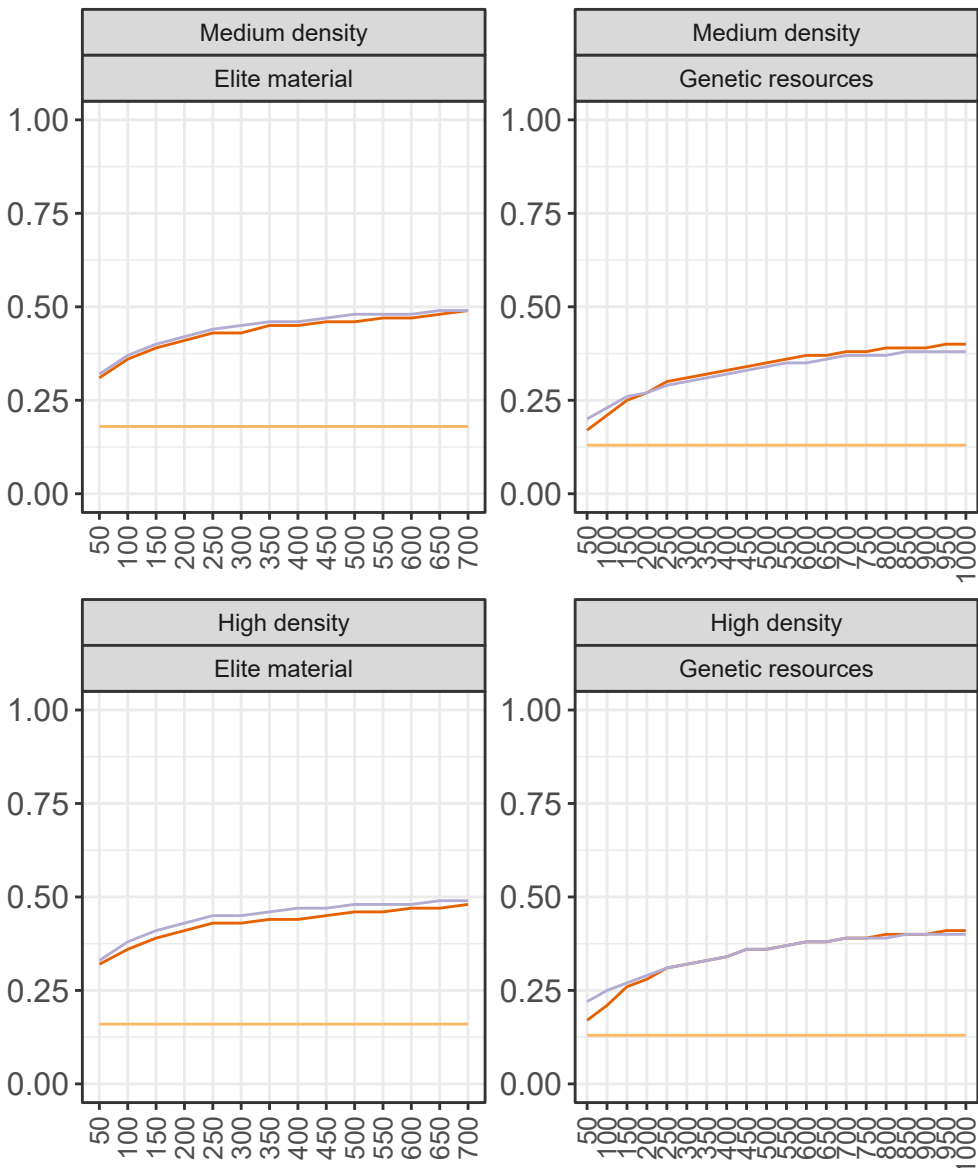

**Method**

- WP<sub>inc</sub>
- AP
- Comb<sub>inc</sub>

Supplement: jkab420_Supplementary_Figures [file jkab420_supplementary_figures.zip › jkab420_Supplementary_Figures/Figure_S20.pdf]

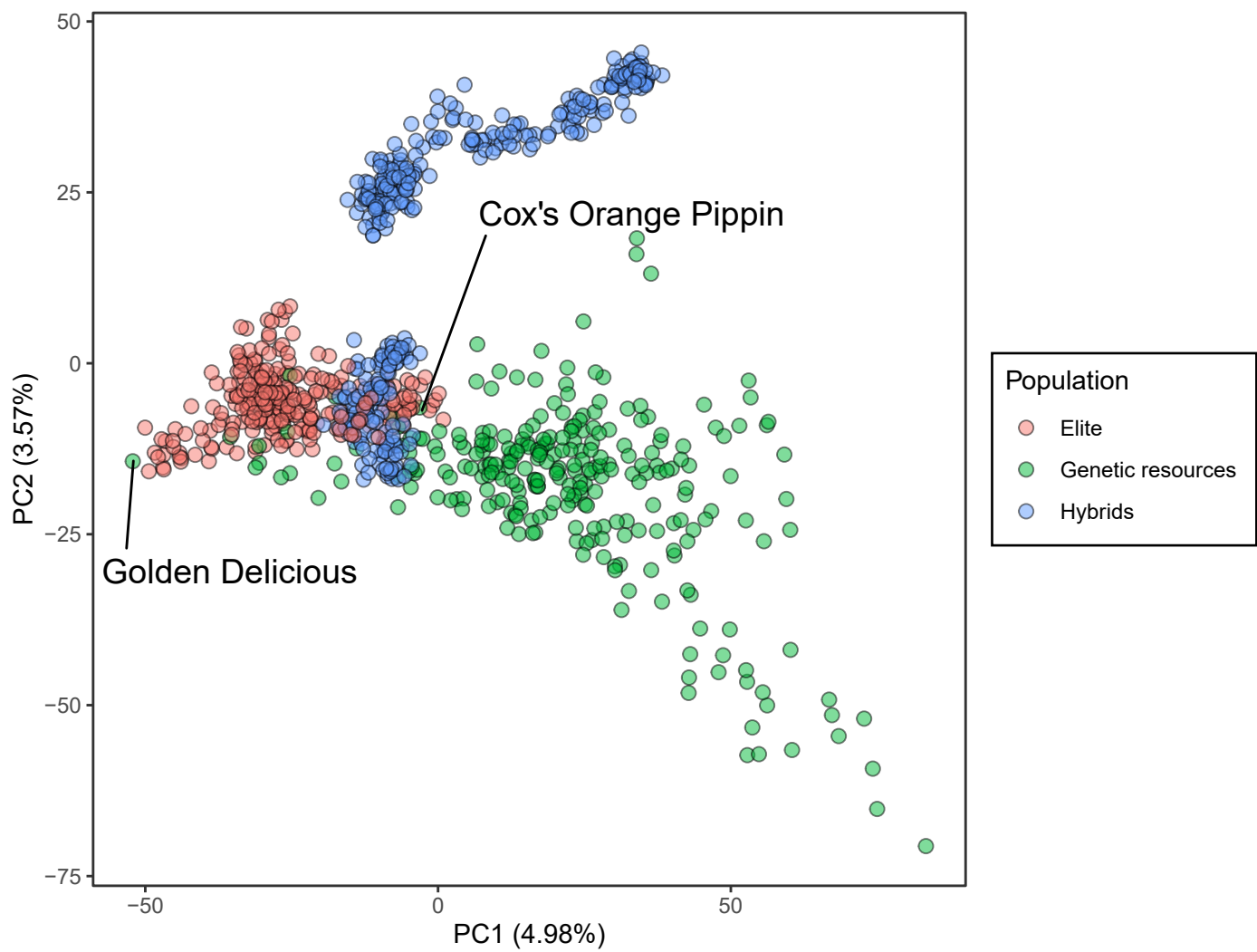

Supplement: jkab420_Supplementary_Figures [file jkab420_supplementary_figures.zip › jkab420_Supplementary_Figures/Figure_S23.pdf]

FBo-Hi

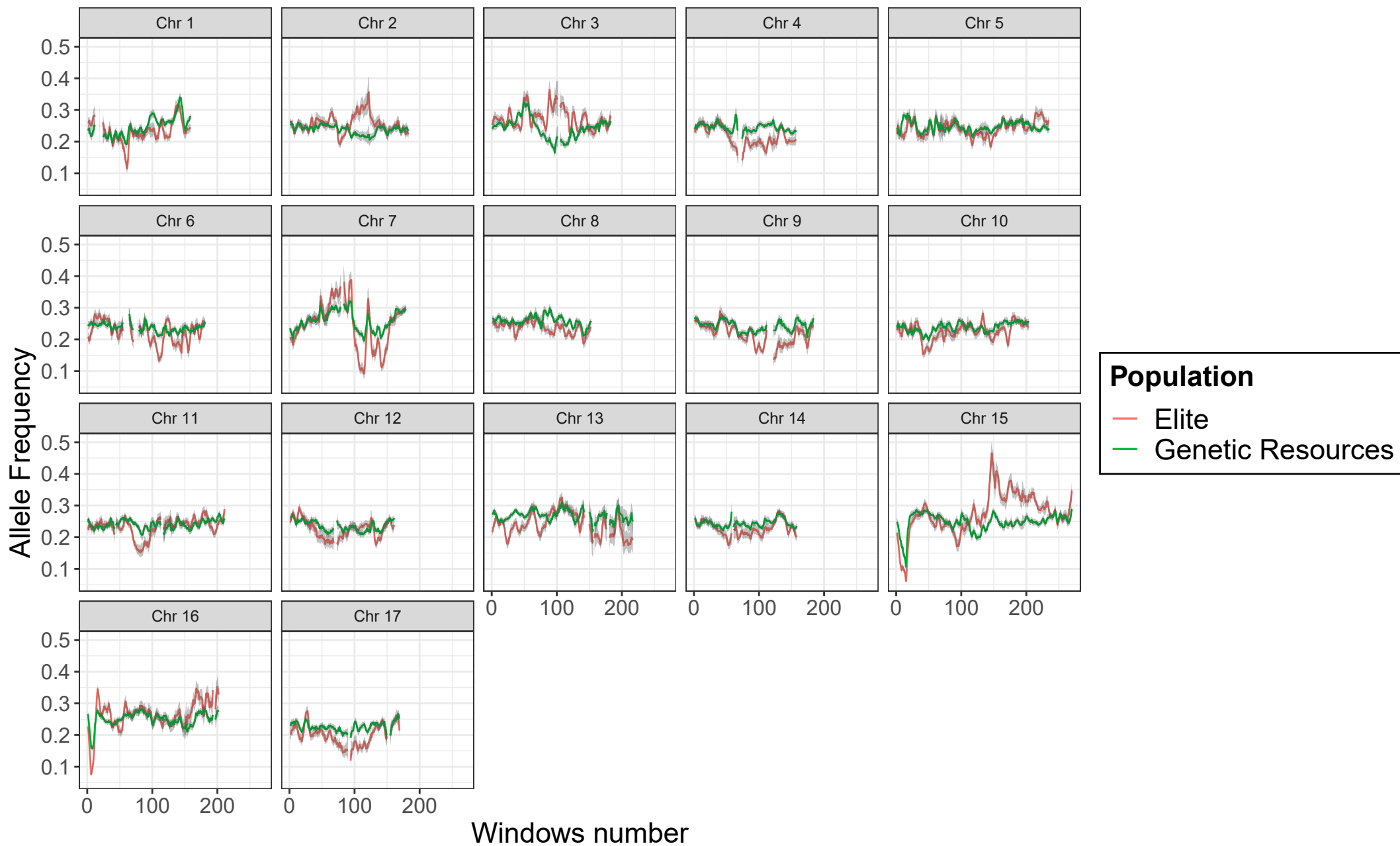

Supplement: jkab420_Supplementary_Figures [file jkab420_supplementary_figures.zip › jkab420_Supplementary_Figures/Figure_S3.pdf]

REFPOP

Allele Frequency

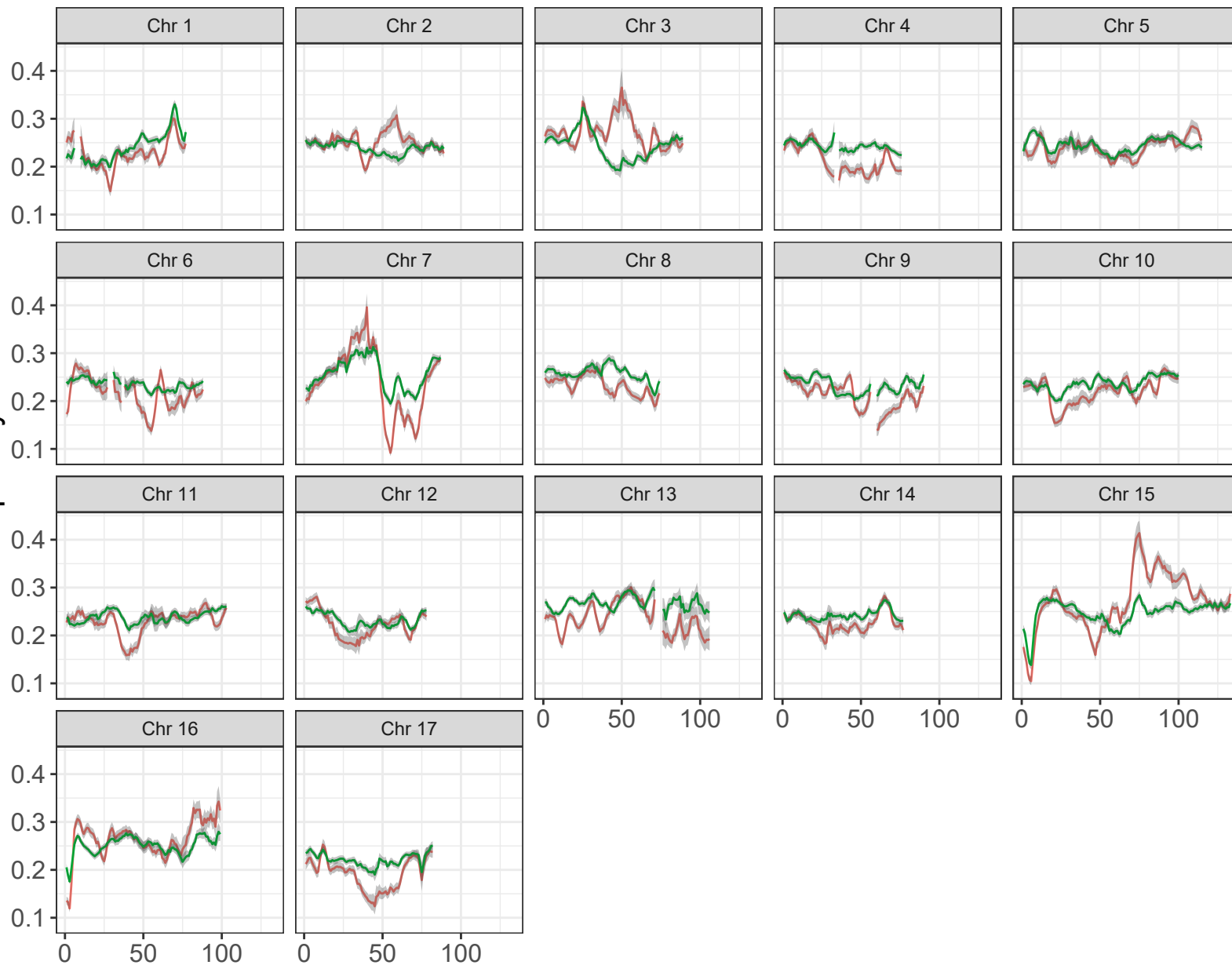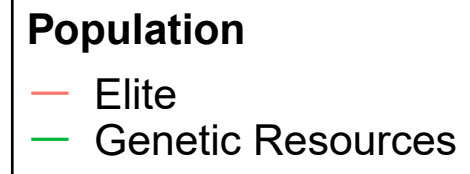

Windows number

Supplement: jkab420_Supplementary_Figures [file jkab420_supplementary_figures.zip › jkab420_Supplementary_Figures/Figure_S4.pdf]

A

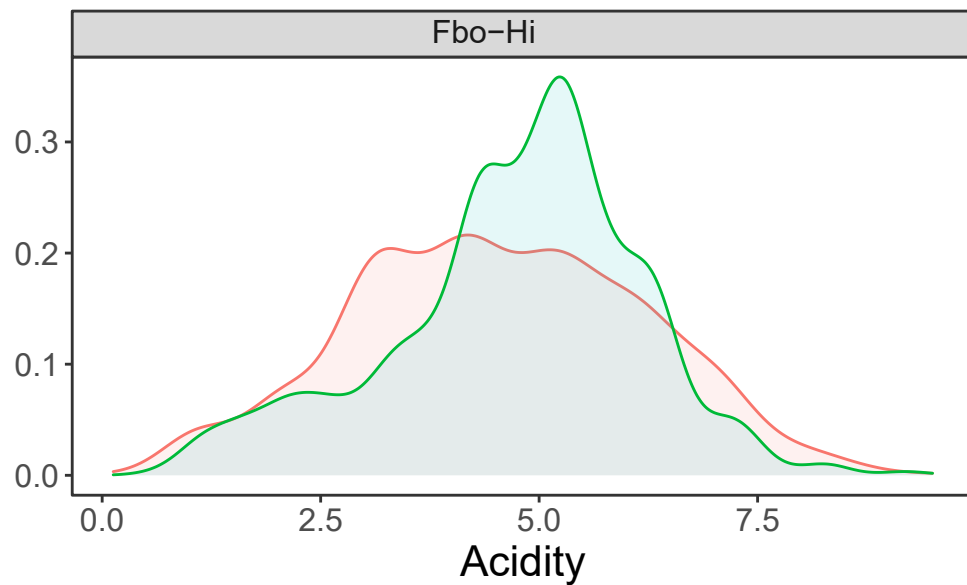

B

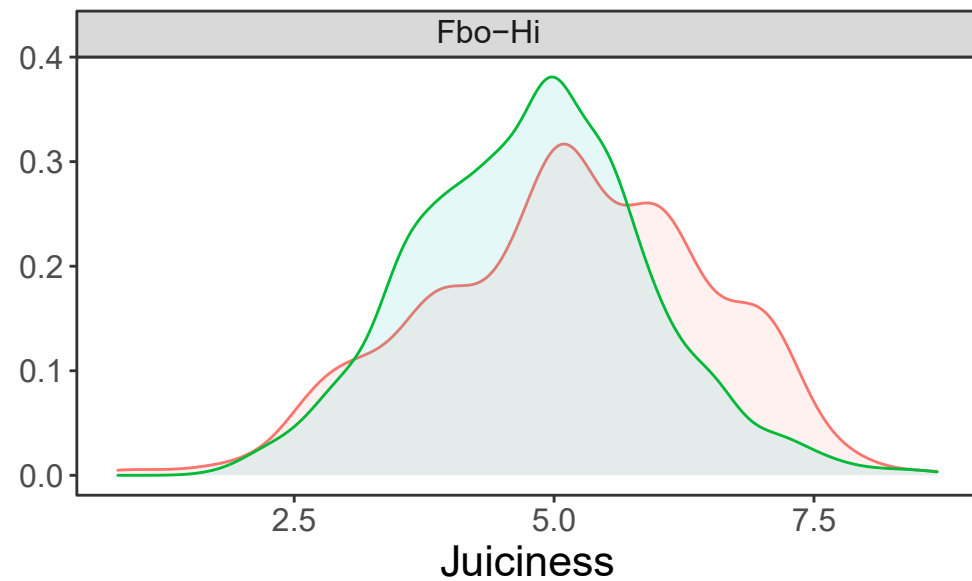

C

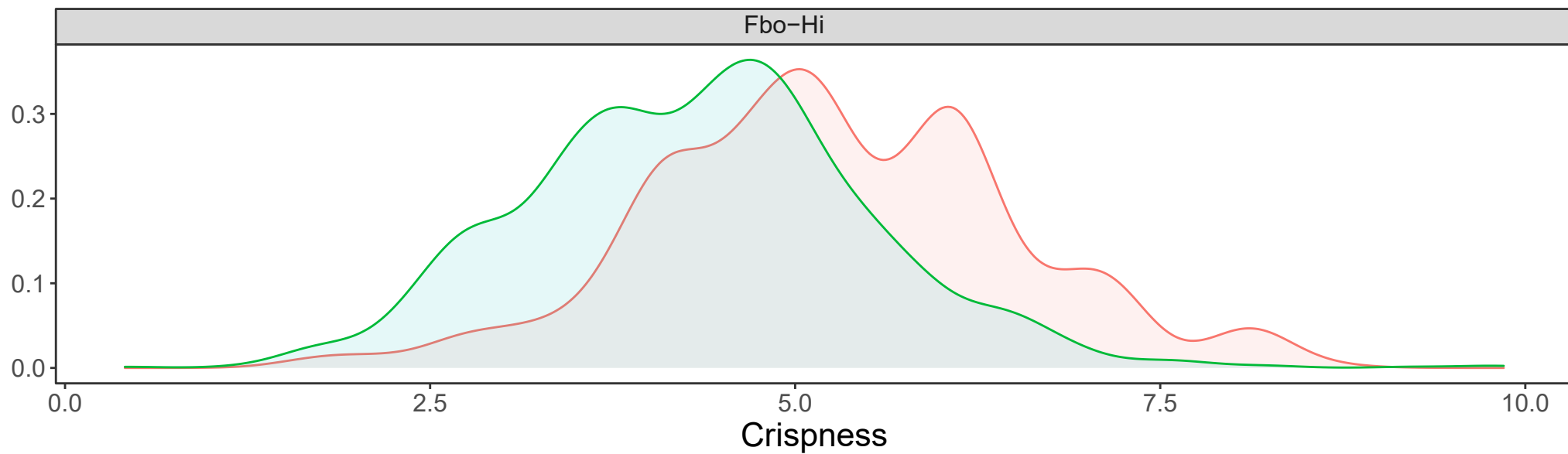

**Population** Elite Genetic Resources

Supplement: jkab420_Supplementary_Figures [file jkab420_supplementary_figures.zip › jkab420_Supplementary_Figures/Figure_S5.pdf]

A

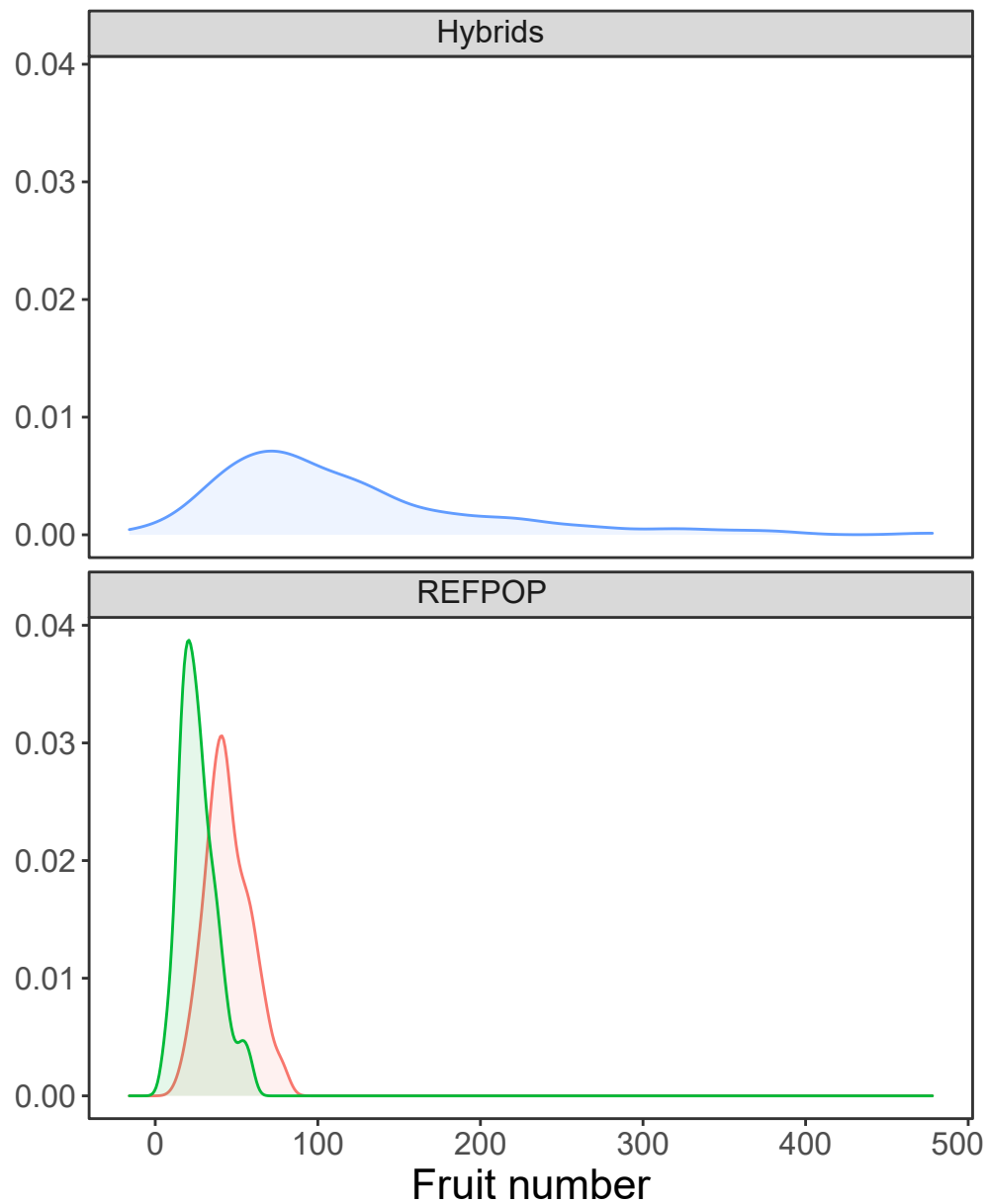

B

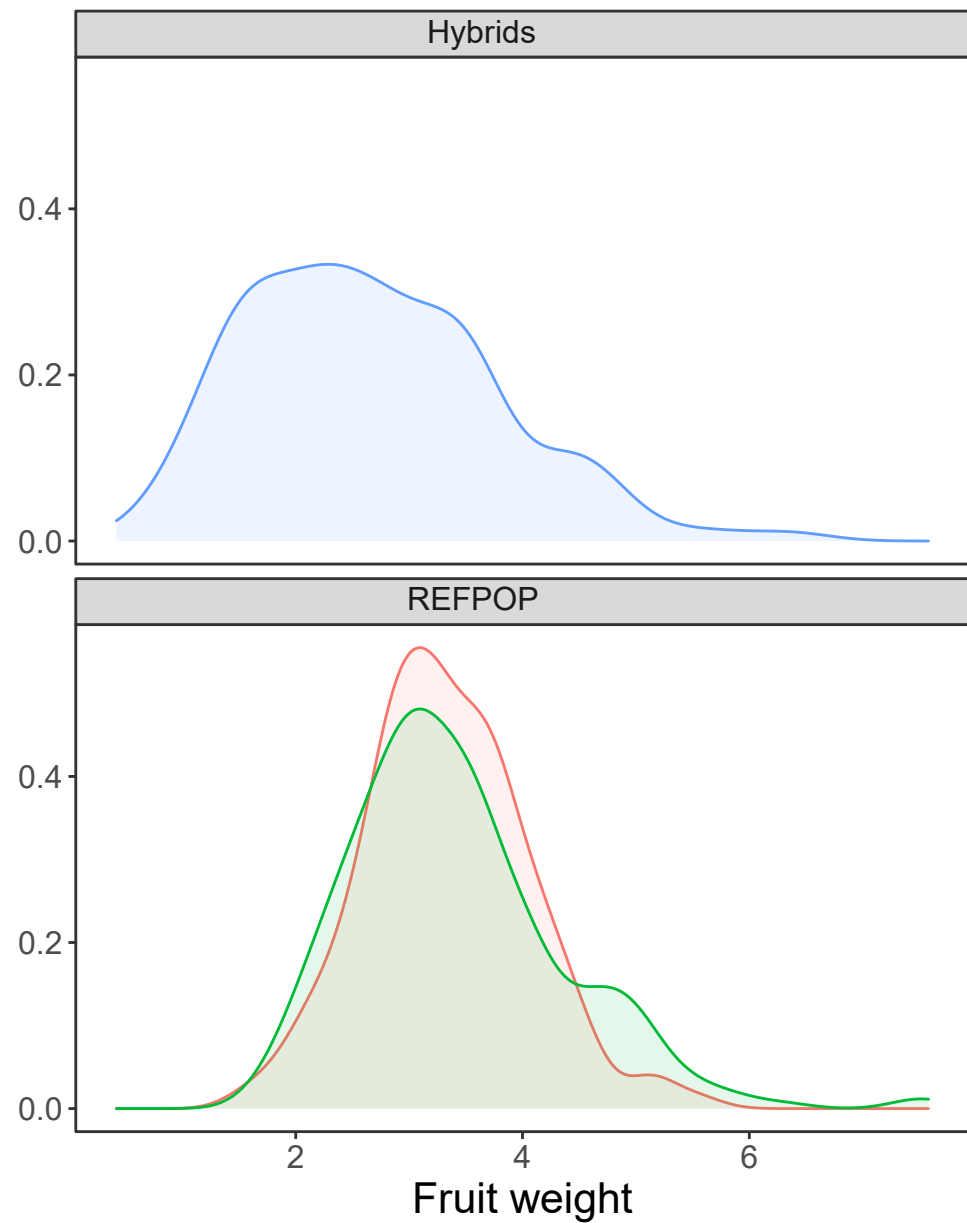

**Population** Elite Genetic Resources Hybrids

Supplement: jkab420_Supplementary_Figures [file jkab420_supplementary_figures.zip › jkab420_Supplementary_Figures/Figure_S6.pdf]

A

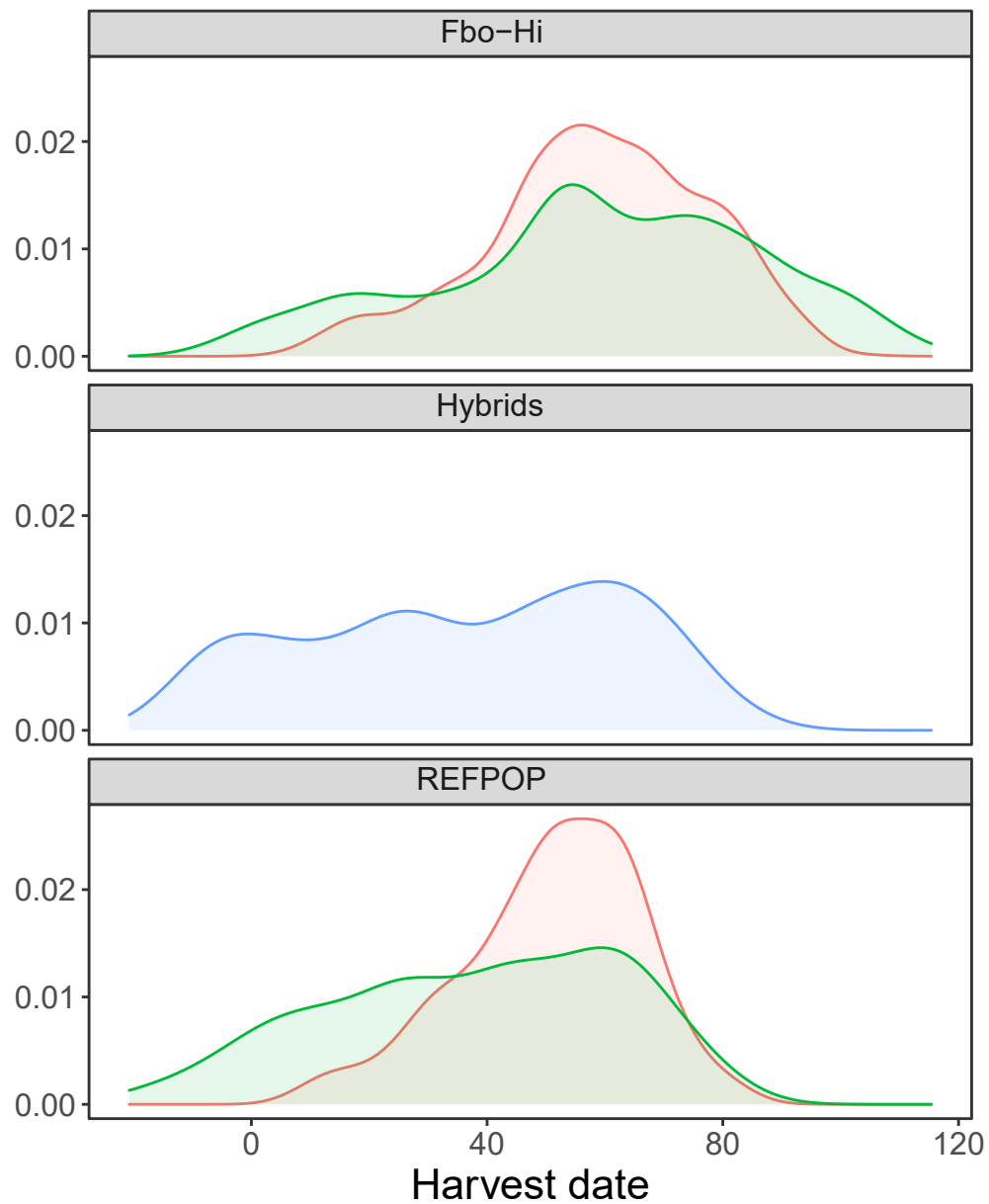

B

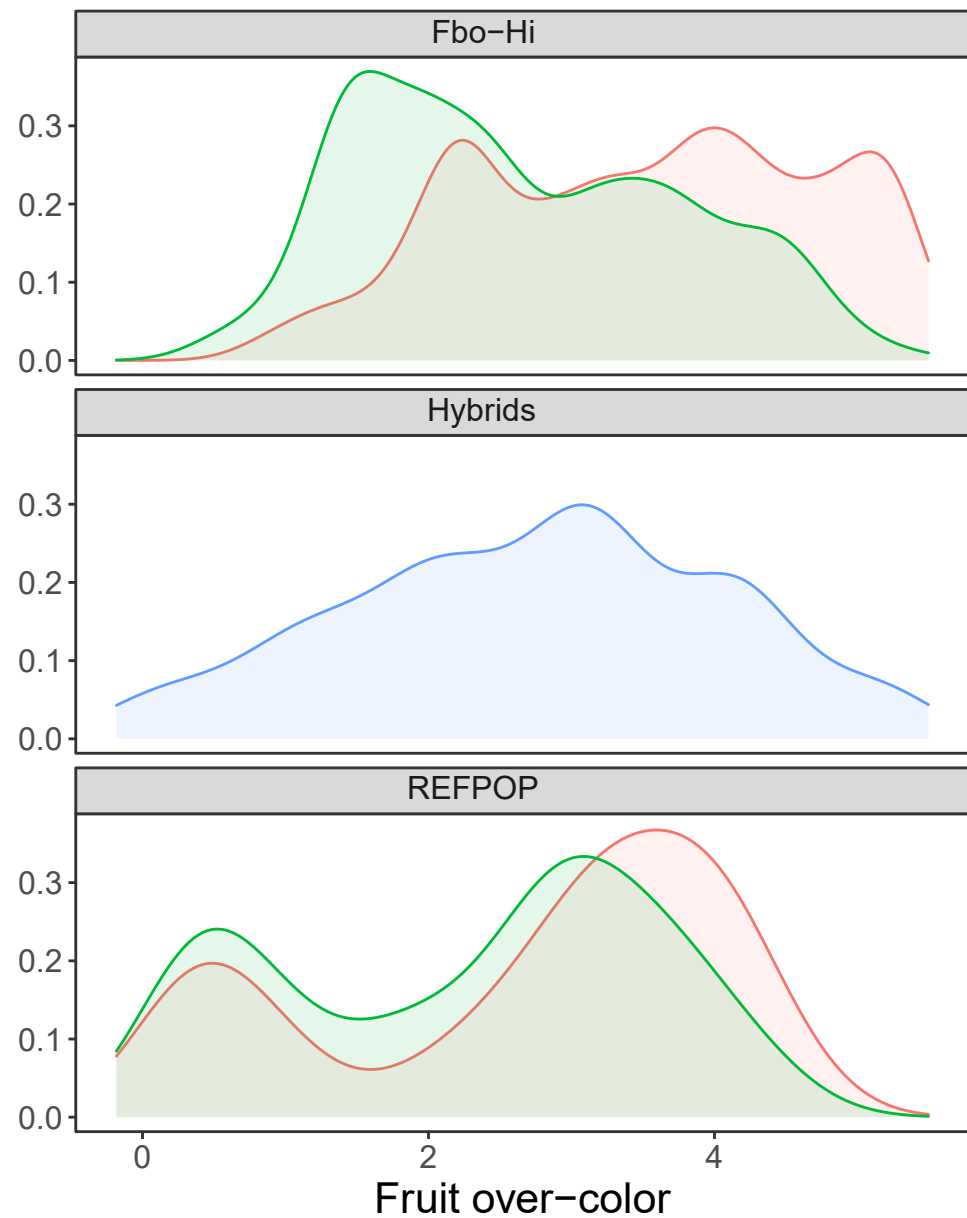

**Population** Elite Genetic Resources Hybrids

Supplement: jkab420_Supplementary_Figures [file jkab420_supplementary_figures.zip › jkab420_Supplementary_Figures/Figure_S7.pdf]

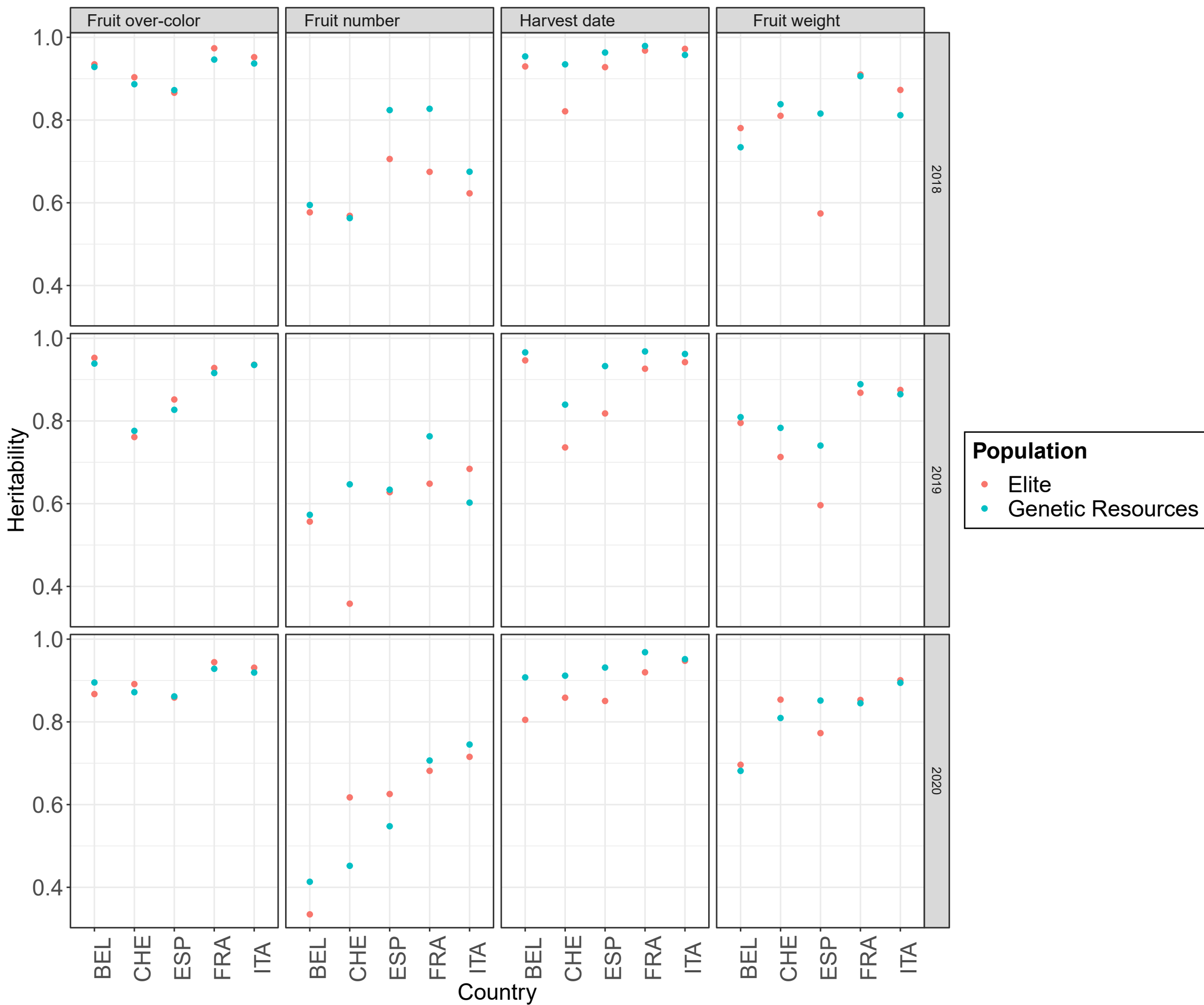

Supplement: jkab420_Supplementary_Figures [file jkab420_supplementary_figures.zip › jkab420_Supplementary_Figures/Figure_S8.pdf]

# Acidity

Fbo-Hi

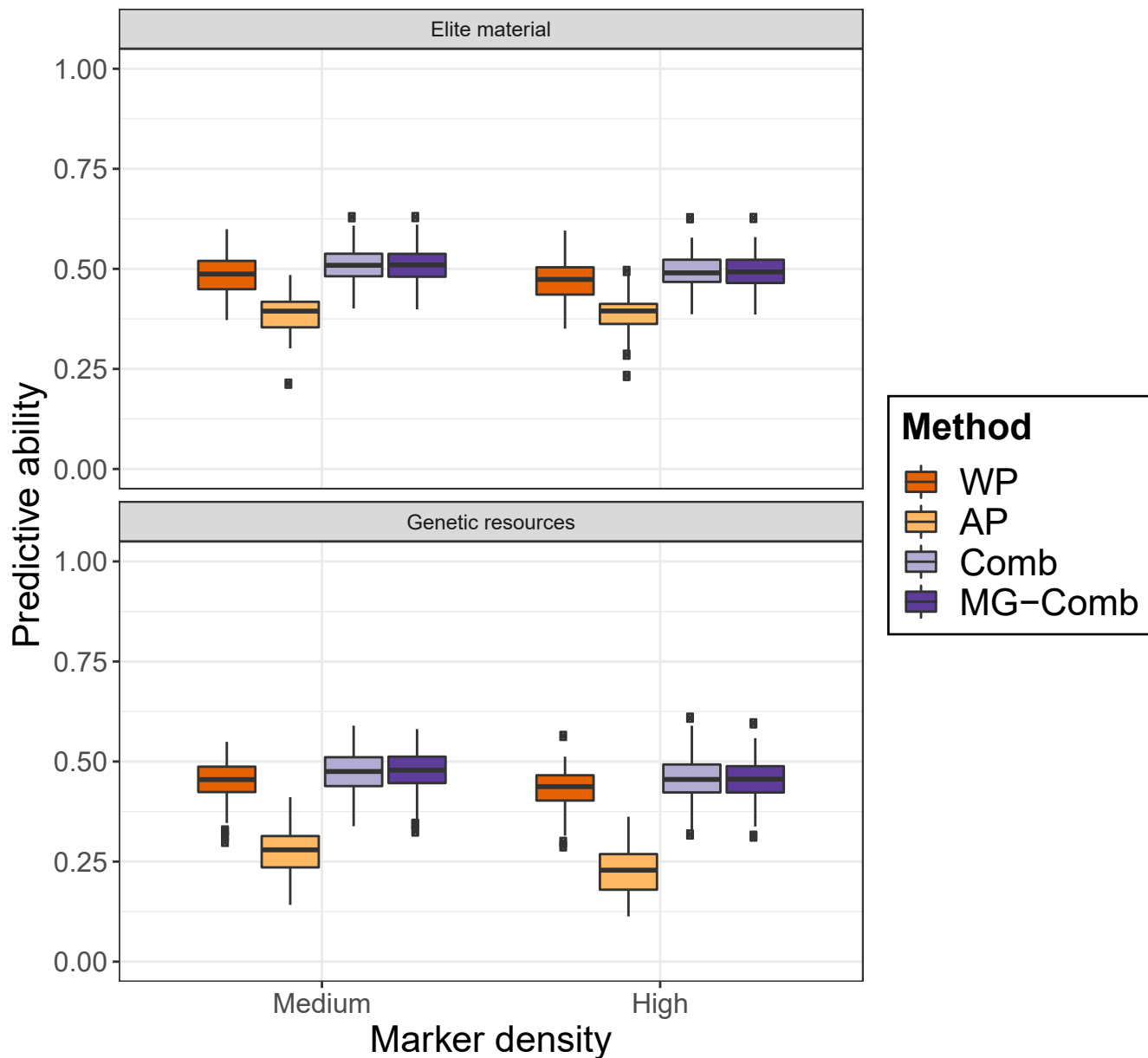

Supplement: jkab420_Supplementary_Figures [file jkab420_supplementary_figures.zip › jkab420_Supplementary_Figures/Figure_S9.pdf]
